# Supplementary material for: Rational design of West Nile virus vaccine through large replacement of 3′ UTR with internal poly(A)
Source: EMBO Mol Med. 2021 Aug 5;13(9):e14108. doi: 10.15252/emmm.202114108 (PMC8422072; doi:10.15252/emmm.202114108)
Supplement: Supplementary file 2 — Appendix [file EMMM-13-e14108-s004.pdf]

Appendix

Rational design of West Nile virus vaccine through large replacement of 3' UTR with internal poly(A)

Ya-Nan Zhang[1] †, Na Li[1]†, Qiu-Yan Zhang[1]†, Jing Liu[1], Shun-Li Zhan[2], Lei Gao[2], Xiang-Yue Zeng[1], Fang Yu[1], Hong-Qing Zhang[1], Xiao-Dan Li[1], Cheng-Lin Deng[1], Pei-Yong Shi[3], Zhi-Ming Yuan[1], Shao-Peng Yuan[2], Han-Qing Ye[1]*, Bo Zhang[1]*

Appendix Figure S1-9
Appendix Tables S1-7

Appendix Figure S1

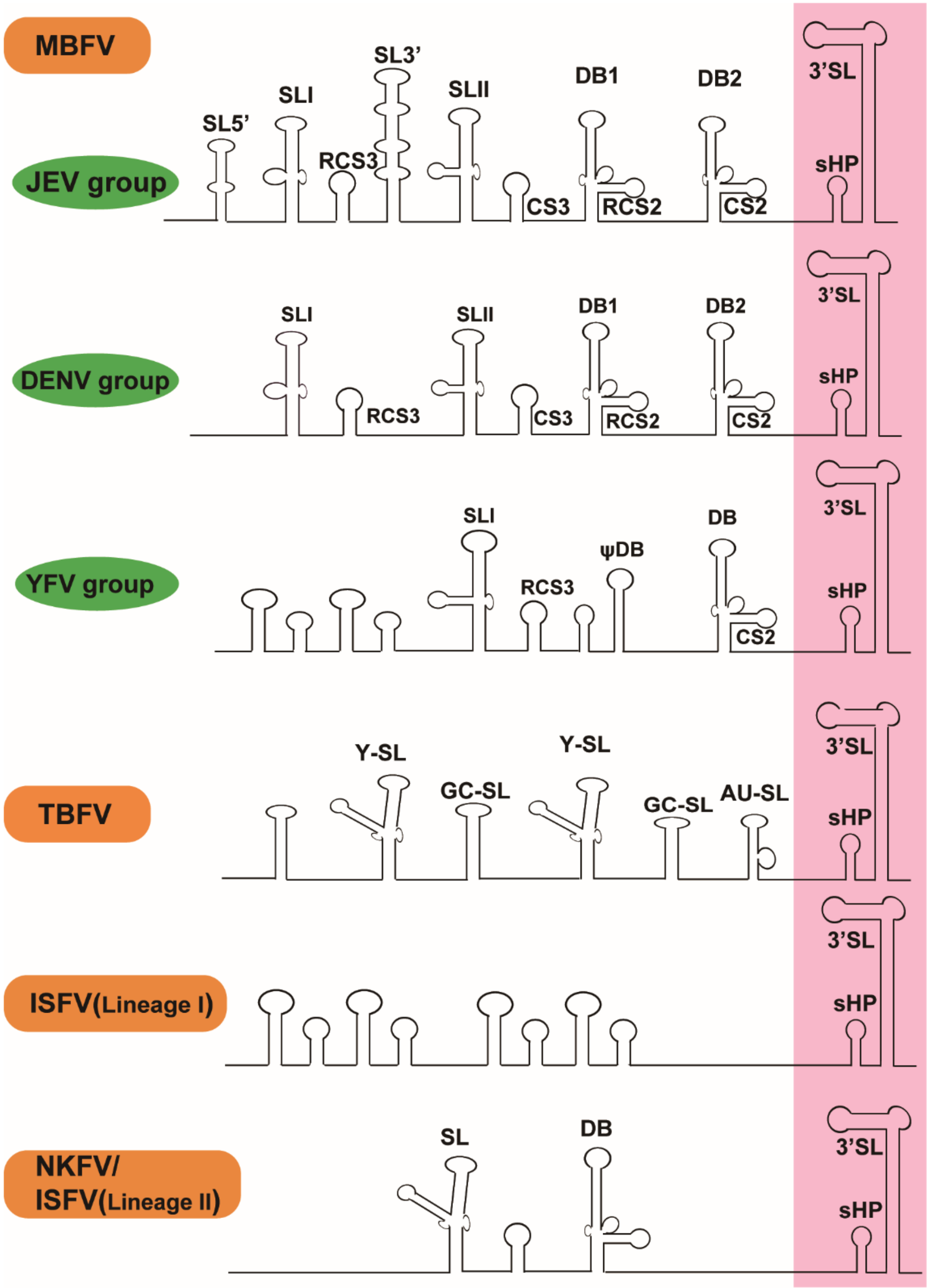

Appendix Figure S1. Diagram of flavivirus 3'UTR secondary structure. Flaviviruses are divided into four groups in terms of transmission vectors: mosquito-borne flaviviruses (MBFV), tick-borne flaviviruses (TBFV), insect specific flaviviruses (ISFV) and non-known flaviviruses (NKFV). In MBFV, the JEV and DENV serological groups contain similar stem loop (SL) and dumbbell (DB) structure in 3'-UTR; YFV group contains simple SL region. In TBFV, there are Y-SL, AU-SL, and GC-SL structures within 3'-UTR. Classical ISFV has multiple small SL, and NKFV possesses a set

of SL and DB structures similar to MBFV. sHP-3′-SL region is the most conservative region in all flaviviruses because of its crucial role in viral genome cyclization.

Appendix Figure S2

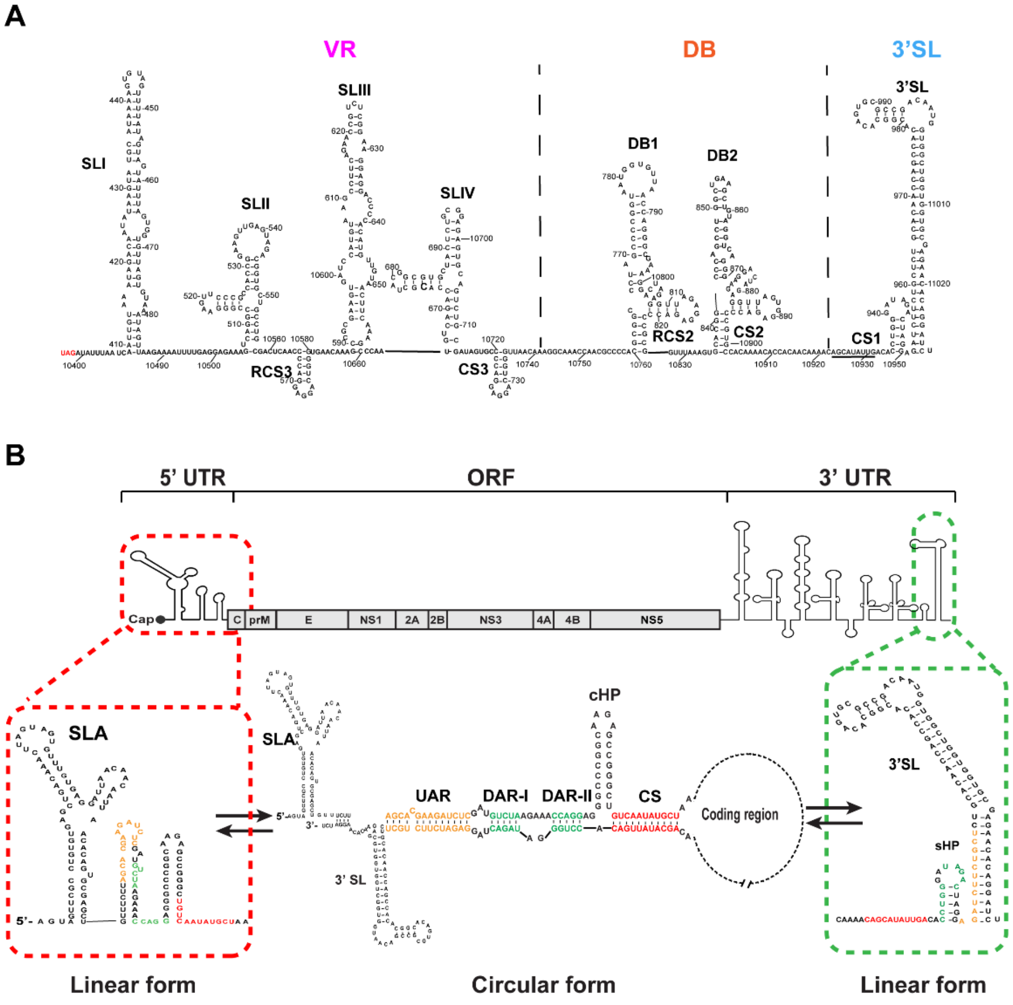

Appendix Figure S2. Schematic illustration of WNV genome.

A. Secondary structure of WNV 3'-UTR.

B. Structures of the linear and circular forms of the WNV genome. The conserved RNA structures in the 5' and 3' terminus of the genome are indicated, and the UAR, DAR-I, DAR-II and CS sequences are highlighted in orange, green, and red, respectively, in the linear genome. The pairwise hybridization between 5'UAR and 3'UAR, 5'DAR and 3'DAR, and 5'CS and 3'CS are shown in the circular form of the genome.

Appendix Figure S3

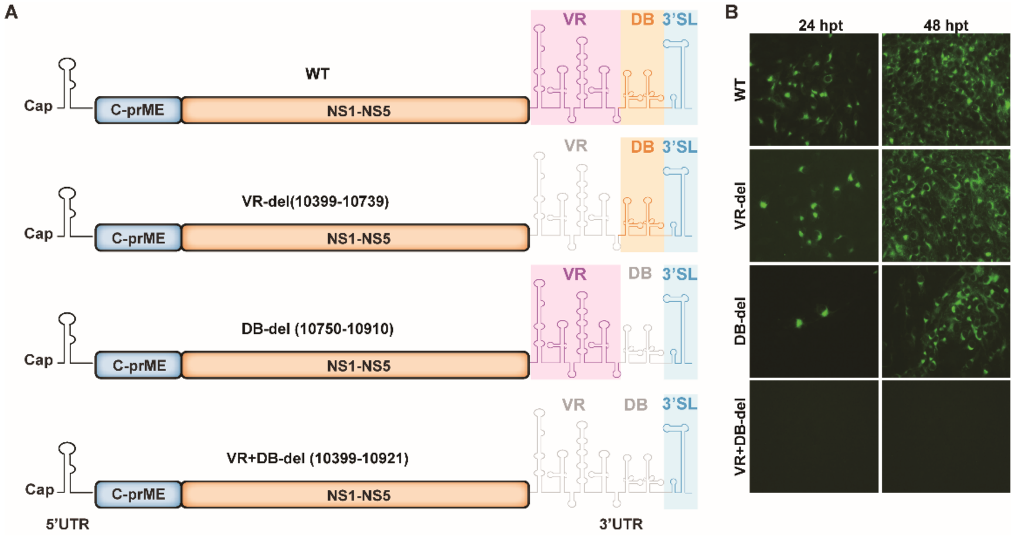

Appendix Figure S3. Characterization of 3'-UTR mutants harboring different deletions of RNA elements

A. Schematic diagram of WT, SL-del, DB-del, SL+DB-del mutants. Area marked with gray represents deleted sequences.

B. IFA analysis of E protein expression in WT, SL-del, DB-del, or SL+DB-del RNA-transfected BHK-21 cells.

Appendix Figure S4

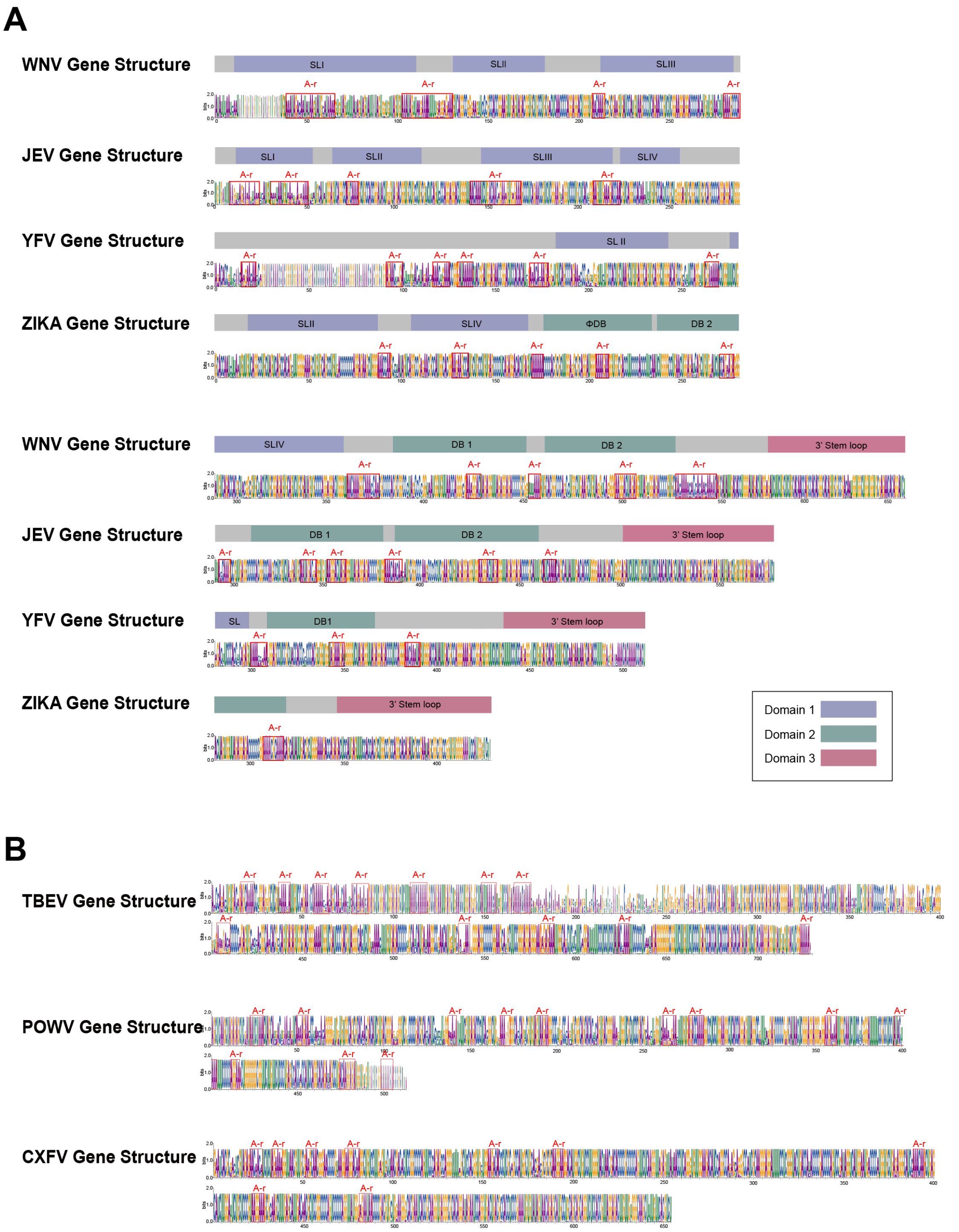

Appendix Figure S4. Alignment of partial flavivirus 3'-UTR sequences.

A. The 3'-UTR genomes of WNV (n=1258), JEV (n=229), YFV (n=211) and ZIKV (n=444) were aligned separately, and the three domains of their genomes are marked with different colors. The conserved stretches were found across 3'-UTRs, corresponding to stem loop (SL), dumbbell (DB) structures and the terminal 3' stem loop (3'SL). They were separated by adenylate-rich (A-r) fragments marked with red boxes. The x-axis of the sequence logos shows the position of the

genome, and the y-axis shows sequence conservation and nucleotide composition. Nucleotides are color coded (blue, cytosine; green, uracil; yellow, guanine; purple, adenine).

B. The 3'-UTR genomes of TBEV (n=158), POWV (n=45) and CXFV (n=27) were aligned separately.

Appendix Figure S5

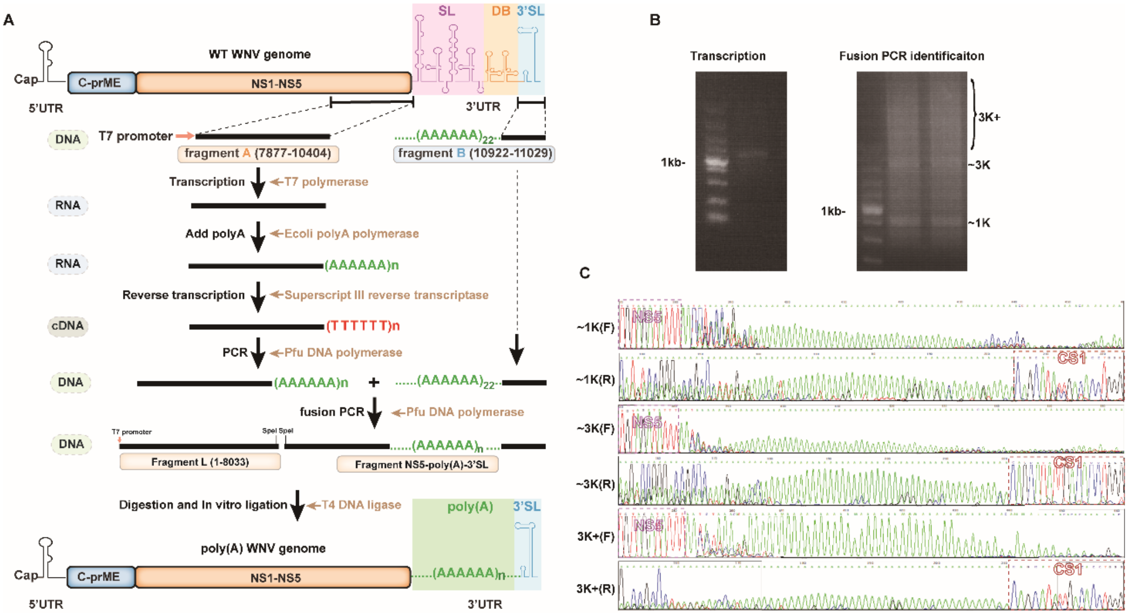

Appendix Figure S5. Construction of the infectious clone of WNV with internal poly(A) insertion.

A. Flow chart of internal poly(A) insertion within infectious clone of WNV. Firstly, fragment A which covered nearly the whole NS5 gene and 6 nt at start from 3'-UTR was amplified from pACYC-WT-WNV template and inserted into pMD18T vector following transcribed into RNAs and added poly(A) tail in vitro. Then, RNAs with poly(A) tail were reverse transcribed into cDNA and subjected to fusion PCR with 3'SL-sequence also amplified from pACYC-WT-WNV template, producing a long fragment named as NS5-polyA-3'SL. Finally, SpeI digested sequence of NS5-polyA-3'SL and PCR amplified long genome with T7 promoter was ligated in vitro to generate WNV-poly(A) infectious clone.

B. Left: Identification of in vitro transcriptional RNA of NS5 fragments. Right: Long DNA fragment of fusion PCR. Different lengths (1Kb, ~3Kb, 3Kb+) of DNA fragments were obtained. C. Sequencing of different fusion DNA fragments. Poly(A) sequences were obtained when sequencing using forward or adverse primers.

Appendix Figure S6

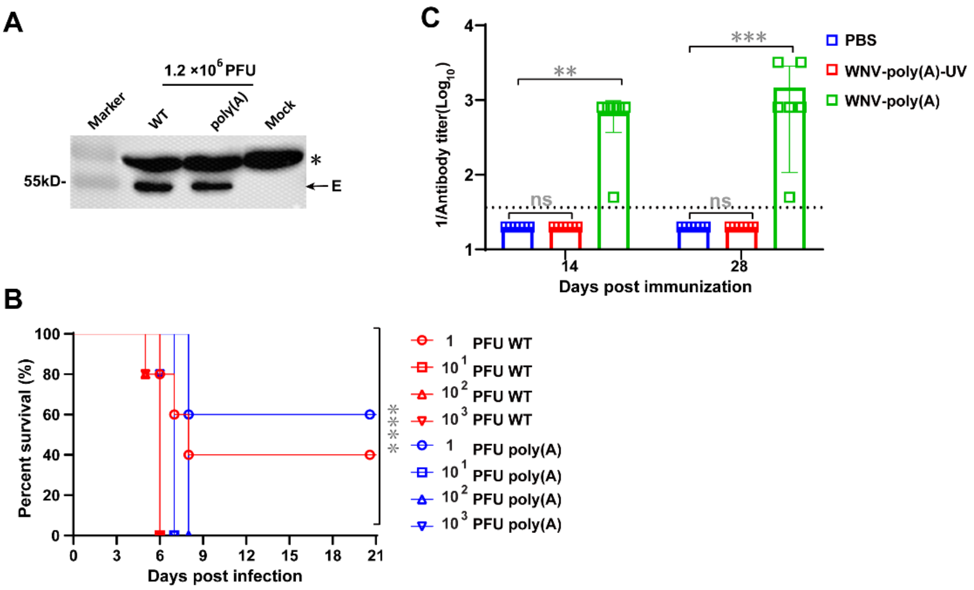

Appendix Figure S6. Characterization of WNV-poly(A).

A. Western Blotting assay for quantification of the levels of viral envelope protein in equal amounts (10^6 PFU) of WT and WNV-poly(A) viruses using anti-Envelope polyclonal antibody. * represents non-specific signal.

B. Virulence study of WNV-poly(A). 4-week-old ICR mice (n=5/per group) were intracranially infected with different dosages of WNV-poly(A) or WT viruses and survival were monitored during 21 day-observation period. Log-rank test were used for survival analysis. ****$p < 0.0001$.

C. Total anti-WNV IgG antibody of WNV-poly(A) and UV- inactivated WNV-poly(A). Six-week-old C57BL/6 (n=5/per group) mice were i.p immunized with 10^5 PFU of both WNV-poly(A) and UV-inactivated virus, respectively, and PBS immunized mice as the negative control. At 14 and 28 days after immunization, sera of mice were collected for ELISA assay. The data represent mean ± sd in each group and the horizontal dotted line represents the limit of detection. Student's T-tests were used to determine statistical analysis. ** $p < 0.01$, *** $p < 0.001$, ns represents not significant.

Appendix Figure S7

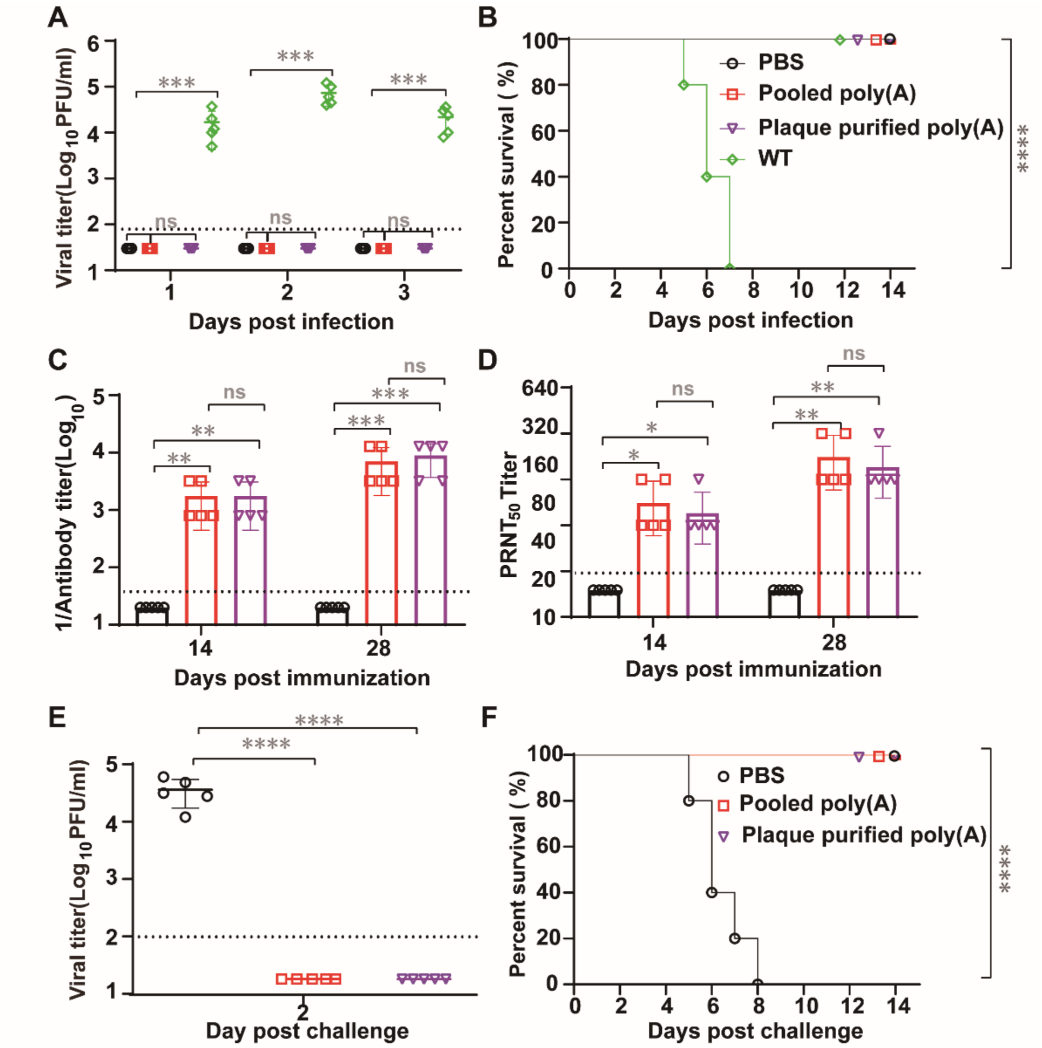

Appendix Figure S7. Plaque purified WNV-poly(A) for virulence and immunization study in C57BL/6 mice.

A-B. Virulence study. Four to six-week-old C57BL/6 mice (n=5 per group) were injected i.p. with 10^7 PFU of pooled WNV-poly(A), plaque purified WNV-poly(A) or PBS (negative control) in a volume of 200 μL. Viremia was quantified by plaque assay from days 1 to 3 post-infection. (The values represent mean ± sd in each group, each symbol represents a mouse. ***p< 0.001, ns represents not significant by one-way ANOVA analysis) (A) and survival was monitored daily till the end of experiment. (****p< 0.0001 by Log-rank test) (B).

C-F. Immunization study. Total anti-WNV IgG antibody (C) and neutralizing antibody titers (D) in mouse serum on the indicated days post immunization were determined. (For C and D, the values represent mean ± sd in each group and the horizontal dotted line represents the limit of detection. *p< 0.05,**p<0.01, *** p<0.001, ns represents not significant by one-way ANOVA analysis).

On day 30 post-immunization, mice were challenged i.p. with 3× 10^7 PFU of WT WNV. Viremia was quantified by plaque assay on day 2 post-challenge. (The values represent mean ± sd in each group, each symbol represents a mouse. ****p< 0.0001, ns represents not significant by one-way ANOVA analysis) (E). Survival was monitored daily for two weeks (****p< 0.0001 by Log-rank test) (F).

Appendix Figure S8

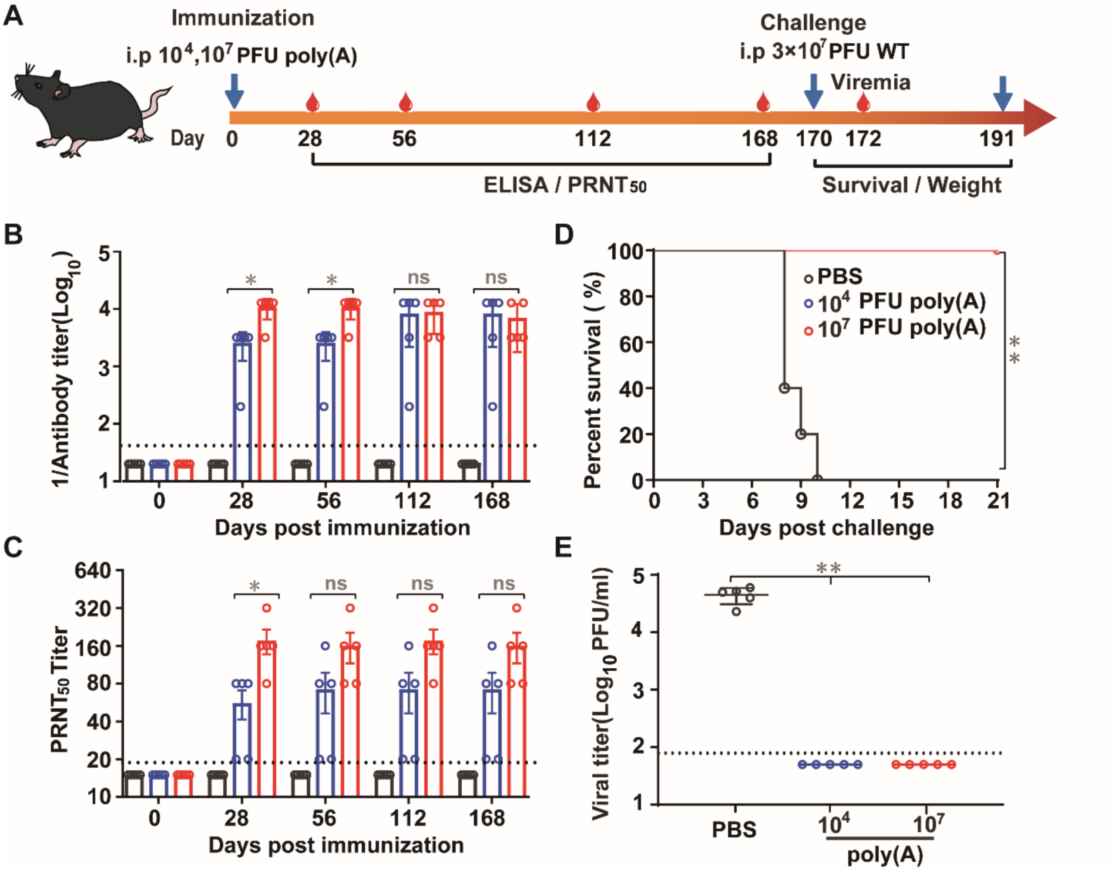

Appendix Figure S8. Long-term efficacy of a single-shot WNV-poly(A) vaccine in C57BL/6 mice.

A. Experimental scheme. Groups of four to six-week-old C57BL/6 mice (n=5 per group) were immunized i.p. with 10^4 or 10^7 PFU of WNV-poly(A) or equal volume of PBS (negative control).

B-C. Total anti-WNV IgG antibody (B) and $PRNT_{50}$ titers (C) were detected at 28, 56, 112, and 168 days post immunization. (For B and C, the values represent mean ± sd in each group and the horizontal dotted line represents the limit of detection. *p< 0.05, **p<0.01, ns represents not significant by Mann–Whitney test).

D. Survival was monitored daily after challenged i.p. with 3×10^7 PFU of WT WNV on day 170 post- immunization. (**p< 0.01 by Log-rank test)

E. Viremia was measured by plaque assay on day 2 post-challenge. (The values represent mean ± sd in each group, each symbol represents a mouse. **$p < 0.01$, ns represents not significant by one-way ANOVA analysis)

Appendix Figure S9

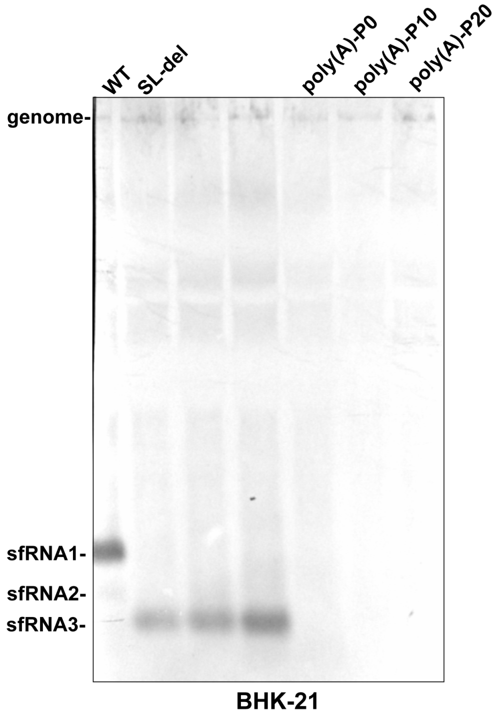

Appendix Figure S9. sfRNA production of WT WNV and WNV-poly(A). Northern blot detection of extracted RNA from BHK-21 cells infected with WT-WNV and mutant of SL-del, poly(A)-P0, poly(A)-P10, poly(A)-P20 at 36 hours post infection. Bands of sfRNA are indicated as sfRNA1, sfRNA2 and sfRNA3 from top to bottom.

Appendix Table S1-7

Table S1A. Nucleotide composition and identity in the three domains of WNV 3'UTRs.

3' UTR	Sequences	Length (bp)		Nucleotide composition (%)					Identical sites		Average identity
Region	n	Mode	Range	A	G	C	U	GC	n	(%)	(%)
Domain I	1258	336	315-356	29.10	27.40	20.10	23.40	47.60	130	36.00	93.80
Domain II	770	188	157-188	31.00	26.30	29.30	13.40	55.60	114	61.00	95.60
Domain III	474	107	70-107	26.10	26.50	28.90	18.50	55.40	75	70.10	99.00

Table S1B. Nucleotide composition and identity of Adenylate-rich spacers in WNV 3'UTRs.

Spacer	Sequences	Length (bp)		Nucleotide composition (%)					Identical sites		Average identity
	n	Mode	Range	A	G	C	U	GC	n	(%)	(%)
SLI	1258	27	25-27	56.60	6.90	11.40	25.10	18.30	0	0.00	89.00
SLI-SLII	1258	28	14-28	52.40	0.10	21.90	25.60	22.00	0	0.00	88.00
SLII-SLIII	1258	7	7	69.90	15.90	14.20	0.10	30.10	5	71.40	97.10
SLIII-SLIV	1258	9	9	54.00	33.30	12.60	0.00	46.00	7	77.80	97.20
SLIV-DB1	770	16	16	62.50	24.00	12.50	1.10	36.50	12	75.00	96.50
DB1	770	7	7	54.80	14.30	30.90	0.00	45.20	5	71.40	96.00
DB1-DB2	770	6	6	55.40	2.70	0.10	41.80	2.80	2	33.30	86.00
DB2	770	10	10	50.00	10.00	30.00	10.00	40.00	9	90.00	99.97
DB2-3'SL	749	17	8-17	70.20	28.70	1.10	0.10	29.80	2	11.80	86.30

Table S2A. Nucleotide composition and identity in the three domains of JEV 3'UTRs.

3' UTR	Sequences	Length (bp)		Nucleotide composition (%)					Identical sites		Average identity
Region	n	Mode	Range	A	G	C	U	GC	n	(%)	(%)
Domain I	229	289	275-289	27.90	23.50	30.10	18.50	53.60	155	53.60	92.50
Domain II	226	182	179-182	29.70	25.30	29.30	15.70	54.60	120	65.90	97.50
Domain III	221	113	70-113	29.30	21.40	27.10	22.30	48.50	46	40.70	98.80

Table S2B. Nucleotide composition and identity of Adenylate-rich spacers in JEV 3'UTRs.

Spacer	Sequences	Length (bp)		Nucleotide composition (%)					Identical sites		Average identity
	n	Mode	Range	A	G	C	U	GC	n	(%)	(%)
SLI	229	16	7-16	54.60	3.00	28.40	14.00	31.40	0	0.00	63.10
SLI	229	20	8-20	55.20	1.10	22.30	21.40	23.40	0	0.00	72.90
SLII	229	7	6-7	71.10	14.40	14.30	0.30	28.70	1	14.30	98.50
SLII-SLIII	229	28	28	52.30	14.30	22.70	10.70	37.00	22	78.60	94.50
SLIII-SLIV	229	15	15	52.10	13.60	21.00	13.40	34.60	9	60.00	97.60
SLIV-DB1	226	7	7	63.30	21.90	14.50	0.30	36.40	3	42.90	92.00
DB1	226	9	9	55.60	11.00	18.00	15.40	29.00	4	44.40	94.20
DB1	226	10	10	50.00	10.00	30.00	10.00	40.00	9	90.00	99.90
DB1-DB2	226	9	9	72.80	21.30	0.80	5.10	22.10	2	22.20	91.50
DB2	226	10	10	50.00	10.00	30.00	10.00	40.00	7	70.00	99.70
DB2-3'SL	226	6	6-7	68.70	15.60	0.20	15.50	15.80	3	42.90	92.10

Table S3A. Nucleotide composition and identity in the three domains of YFV 3’UTRs.

Region	Sequences	Length (bp)		Nucleotide composition (%)					Identical sites		Average identity
	n	Mode	Range	A	G	C	U	GC	n	(%)	(%)
Domain I	211	204	201-275	33.50	26.70	25.20	14.70	51.80	110	40.10	78.50
Domain II	212	122	115-122	28.20	26.30	28.10	17.40	54.40	80	65.60	93.90
Domain III	164	113	84-114	29.10	24.60	23.60	22.80	48.20	86	74.80	98.00

Table S3B. Nucleotide composition and identity of Adenylate-rich spacers in YFV 3'UTRs.

Spacer	Sequences	Length (bp)		Nucleotide composition (%)					Identical sites		Average identity
	n	Mode	Range	A	G	C	U	GC	n	(%)	(%)
Domain I	211	8	4-8	70.80	9.30	16.70	3.10	26.10	3.00	37.50	84.90
Domain I	211	9	9	50.70	15.30	27.00	7.10	42.30	3	33.30	87.60
Domain I	211	10	10	69.10	9.60	14.50	6.80	24.00	3.00	30.00	86.80
Domain I	211	7	7	85.40	0.00	0.20	14.40	0.20	6.00	85.70	99.50
Domain I	211	10	9-10	67.30	14.50	17.70	0.50	32.30	5.00	50.00	88.80
SL II-SL	211	7	6-7	71.30	14.40	14.40	0.00	28.70	7	100.00	100.00
SL-DB1	212	9	7-9	80.50	13.20	5.30	1.00	18.50	2	22.20	74.00
DB1	212	7	7	70.40	0.00	29.60	0.00	29.60	5	71.40	98.00
DB1-3’SL	212	8	6-8	73.90	11.40	0.40	14.40	11.80	3	37.50	91.90

Table S4A. Nucleotide composition and identity in the three domains of ZIKV 3'UTRs.

Region	Sequences	Length (bp)		Nucleotide composition (%)					Identical sites		Average identity
	n	Mode	Range	A	G	C	U	GC	n	(%)	(%)
Domain I	444	168	164-168	25.50	28.90	29.20	16.40	58.10	80	47.60	97.10
Domain II	374	151	129-151	29.10	26.20	29.70	15.00	56.00	118	78.10	98.30
Domain III	173	109	79-110	24.40	28.10	31.50	16.00	59.60	86	78.20	99.20

Table S4B. Nucleotide composition and identity of Adenylate-rich spacers in ZIKV 3'UTRs.

Spacer	Sequences	Length (bp)		Nucleotide composition (%)					Identical sites		Average identity
	n	Mode	Range	A	G	C	U	GC	n	(%)	(%)
SLII-SLIV	444	7	7	71.00	28.20	0.00	0.80	28.20	2	28.60	98.20
SIV	444	9	9	55.60	22.20	22.20	0.00	44.40	9	100.00	100.00
SIV-ΦDB	374	7	7	85.70	14.30	0.00	0.00	14.30	6	85.70	99.96
ΦDB	374	7	7	85.70	0.00	14.30	0.00	14.30	6	85.70	99.90
DB2	374	7	7	52.70	18.30	28.90	0.00	47.20	4	57.10	93.40
DB2	374	11	11	72.60	18.30	9.10	0.00	27.40	7	63.60	99.70

Table S5. Nucleotide composition and identity in the three domains of TBEV 3'UTRs.

Region	Sequences	Length (bp)	Nucleotide composition (%)					Identical sites		Average identity
	n	Range	A	G	C	U	GC	n	(%)	(%)
3’UTR	158	7	70.4	14.5	15.1	0.00	29.6	0	0.0	89.2
3’UTR	158	6	76.7	5.7	8.2	9.5	13.8	0	0.0	70.6
3’UTR	158	8	76.1	7.1	16.3	0.6	23.4	0	0.0	64.2
3’UTR	158	9	62.2	15.4	17.9	4.4	33.4	0	0.0	41.7
3’UTR	158	9	88.6	0.3	11.1	0.00	11.4	0	0.0	36.9

3'UTR	158	8	62.5	0.0	9.7	27.8	9.7	0	0.0	35.3
3'UTR	158	9	86.4	0.9	12.3	0.4	13.2	0	0.0	34.5
3'UTR	158	7	58.2	24	12.2	5.5	36.3	0	0.0	53.1
3'UTR	158	7	71.1	15.2	5.8	8	20.9	0	0.0	59.6
3'UTR	158	7	66.7	32.4	0.2	0.7	32.5	4	57.1	92.3
3'UTR	158	6	72.5	0.0	24.9	2.6	24.9	5	83.3	89.8
3'UTR	158	6	99.1	0.0	0.9	0.0	0.9	3	50	98.5

Table S6. Nucleotide composition and identity in the three domains of POWV 3'UTRs.

Region	Sequences	Length (bp)	Nucleotide composition (%)					Identical sites		Average identity
	n	Range	A	G	C	U	GC	n	(%)	(%)
3'UTR	45	8	64.2	0.0	11.9	23.8	11.9	8	100.0	100.0
3'UTR	45	8	64.2	0.0	11.9	23.8	11.9	8	100.0	100.0
3'UTR	45	8	66.0	9.4	9.0	15.6	18.4	1	16.7	52.8
3'UTR	45	8	69.9	30.1	0.0	0.0	30.1	4	66.7	89.7
3'UTR	45	8	62.5	25	0.0	12.5	25.0	8	100.0	100.0
3'UTR	45	8	75.6	21.7	2.8	0.0	24.4	3	37.5	75.4
3'UTR	45	8	66.7	11.1	22.2	0.0	33.3	9	100.0	100.0
3'UTR	45	8	78.2	16.0	5.1	0.7	21.1	5	71.4	89.4
3'UTR	45	8	61.9	0.6	15.2	22.3	15.8	6	75.0	94.5
3'UTR	45	8	64.3	35.7	0.0	0.0	35.7	4	66.7	95.6
3'UTR	45	8	59.3	26.7	14.0	0.0	40.7	6	66.7	97.5
3'UTR	45	**8**	85.7	0.0	14.3	0.0	14.3	7	100.0	100.0

Table S7. Nucleotide composition and identity in the three domains of CXFV 3'UTRs.

Region	Sequences	Length (bp)	Nucleotide composition (%)					Identical sites		Average identity
	n	Range	A	G	C	U	GC	n	(%)	(%)
3'UTR	27	7	64.6	1.1	31.7	2.6	32.8	4	57.1	85.5
3'UTR	27	7	78.4	18.5	2.5	0.6	21.0	3	50.0	90.9
3'UTR	27	7	63.6	19.8	16.7	0.0	36.4	5	83.3	94.8
3'UTR	27	7	60.2	25.5	12.5	1.9	38.0	6	75.0	95.8
3'UTR	27	7	64.8	16.7	16.7	1.9	33.3	5	83.3	96.6
3'UTR	27	7	66.7	33.3	0.0	0.0	33.3	6	100.0	100.0
3'UTR	27	7	83.3	0.0	16.7	0.0	16.7	6	100.0	100.0
3'UTR	27	7	71.4	28.6	0.0	0.0	28.6	7	100.0	100.0
3'UTR	27	7	68.1	17.6	0.0	14.3	17.6	6	85.7	94.7
