# Supplementary figures and images for: Rational design of West Nile virus vaccine through large replacement of 3′ UTR with internal poly(A)
Source: EMBO Mol Med. 2021 Aug 5;13(9):e14108. doi: 10.15252/emmm.202114108 (PMC8422072; doi:10.15252/emmm.202114108)

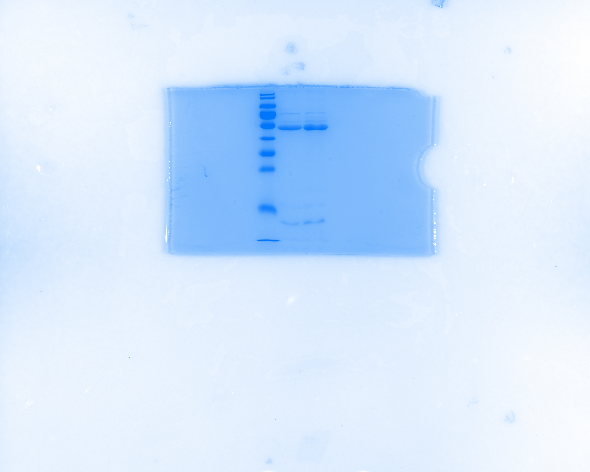

Supplement: Supplementary file 3 — Source Data for Figure 1 [file EMMM-13-e14108-s003.zip › EMM-2021-14108-V3-Figure_1G_Source_Data-sd.tiff]

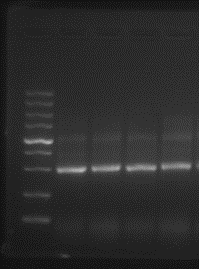

Supplement: Supplementary file 3 — Source Data for Figure 1 [file EMMM-13-e14108-s003.zip › EMM-2021-14108-V3-Figure1D_Source_Data-sd.png]

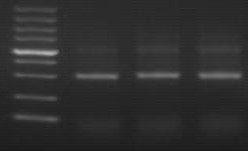

Supplement: Supplementary file 4 — Source Data for Figure 5 [file EMMM-13-e14108-s001.jpg]
